# Polymertropism of rod-shaped bacteria: movement along aligned polysaccharide fibers

**DOI:** 10.1038/s41598-017-07486-0

**Published:** 2017-08-11

**Authors:** David J. Lemon, Xingbo Yang, Pragya Srivastava, Yan-Yeung Luk, Anthony G. Garza

**Affiliations:** 1 Department of Biology, Syracuse University, Syracuse, NY 13244 United States; 2 Department of Physics, Syracuse University, Syracuse, NY 13244 United States; 3 Department of Chemistry, Syracuse University, Syracuse, NY 13244 United States; 4 Department of Physics and Astronomy, Northwestern University, Evanston, IL 60208 United States; 5 The Francis Crick Institute, London, NW1 1BF United Kingdom

## Abstract

In nature, bacteria often live in surface-associated communities known as biofilms. Biofilm-forming bacteria typically deposit a layer of polysaccharide on the surfaces they inhabit; hence, polysaccharide is their immediate environment on many surfaces. In this study, we examined how the physical characteristics of polysaccharide substrates influence the behavior of the biofilm-forming bacterium *Myxococcus xanthus*. *M*. *xanthus* responds to the compression-induced deformation of polysaccharide substrates by preferentially spreading across the surface perpendicular to the axis of compression. Our results suggest that *M*. *xanthus* is not responding to the water that accumulates on the surface of the polysaccharide substrate after compression or to compression-induced changes in surface topography such as the formation of troughs. These directed surface movements do, however, consistently match the orientation of the long axes of aligned and tightly packed polysaccharide fibers in compressed substrates, as indicated by behavioral, birefringence and small angle X-ray scattering analyses. Therefore, we suggest that the directed movements are a response to the physical arrangement of the polymers in the substrate and refer to the directed movements as polymertropism. This behavior might be a common property of bacteria, as many biofilm-forming bacteria that are rod-shaped and motile on soft surfaces exhibit polymertropism.

## Introduction

In nature, bacteria commonly live in surface-associated communities of cells known as biofilms. In recent years many microbiologists have become interested in how the behavior and organization of biofilm-forming bacteria are influenced by the physical characteristics of their substrates^1^. Since biofilms can cause persistent infections when they form on implanted medical devices (for a review, see ref. 2) and can cause structural damage when they form on industrial equipment or pipes^3^, there has been a major effort to identify physical features in manufactured substrates that inhibit biofilm formation and to synthesize novel substrates with biofilm-resistant properties.

Our research has focused on how the physical characteristics of polysaccharide substrates influence the behavior of biofilm-forming bacteria. We have focused on the physical cues in polysaccharide substrates because biofilm-forming bacteria coat the surface to which they are attached with a matrix of polysaccharide^4–6^ and polysaccharide is their immediate environment on many surfaces.

Previous work by Fontes and Kaiser^7^ showed that cells of the biofilm-forming bacterium *Myxococcus xanthus* respond to physical changes in agar, which is a mixture of polysaccharides composed primarily of the linear polymer agarose. In particular, rod-shaped *M*. *xanthus* cells, which move in the direction of their long axes, respond within minutes to compression of their agar substrate by reorienting their long axes perpendicular to the direction of compression. Furthermore, the outward movements of groups of cells from the edges of colonies and the overall expansion of these colonies are primarily oriented perpendicular to the axis of compression. These directed movements require a functional motility system known as the A-motility system, suggesting that the movements result from an active biological process and are not a passive event. However, the specific nature of the physical stimuli to which *M*. *xanthus* cells respond was not uncovered in this study and is addressed in the work described here.

The results of this study suggest that the directed surface movements of *M*. *xanthus* are not a response to the water that accumulates on the surface of the agar after compression or to compression-induced changes in surface topography such as the formation of troughs. We did, however, identify a strong correlation between the strength of *M*. *xanthus*’ response to agar compression and predicted strain, suggesting that the deformation of the agar may dictate the observed changes in the orientation of individual bacterial cells, groups of bacterial cells, and colony spreading. It was previously suggested that *M*. *xanthus* cells may respond to the compression-induced alignment of polysaccharide bundles or fibers in their agar substrate^7^. Indeed, based on the birefringence and small angle X-ray scattering patterns of compressed polysaccharide substrates, and the results of surface spreading assays, we conclude that the directed group movements and expansion on polysaccharide substrates matches the orientation of the long axes of aligned and tightly packed polysaccharide fibers in the substrates. This behavior, which we refer to as polymertropism, might be a common property of bacteria, as many biofilm-forming bacteria that are rod-shaped and motile on soft surfaces are positive for polymertropism. A model for the mechanism of polymertropism, and the potential roles of polymertropism in the behavior and organization of biofilm-forming bacteria are discussed.

## Results

### Compressed polysaccharide substrates promote directed *M. xanthus* movements

**Figure 1:**
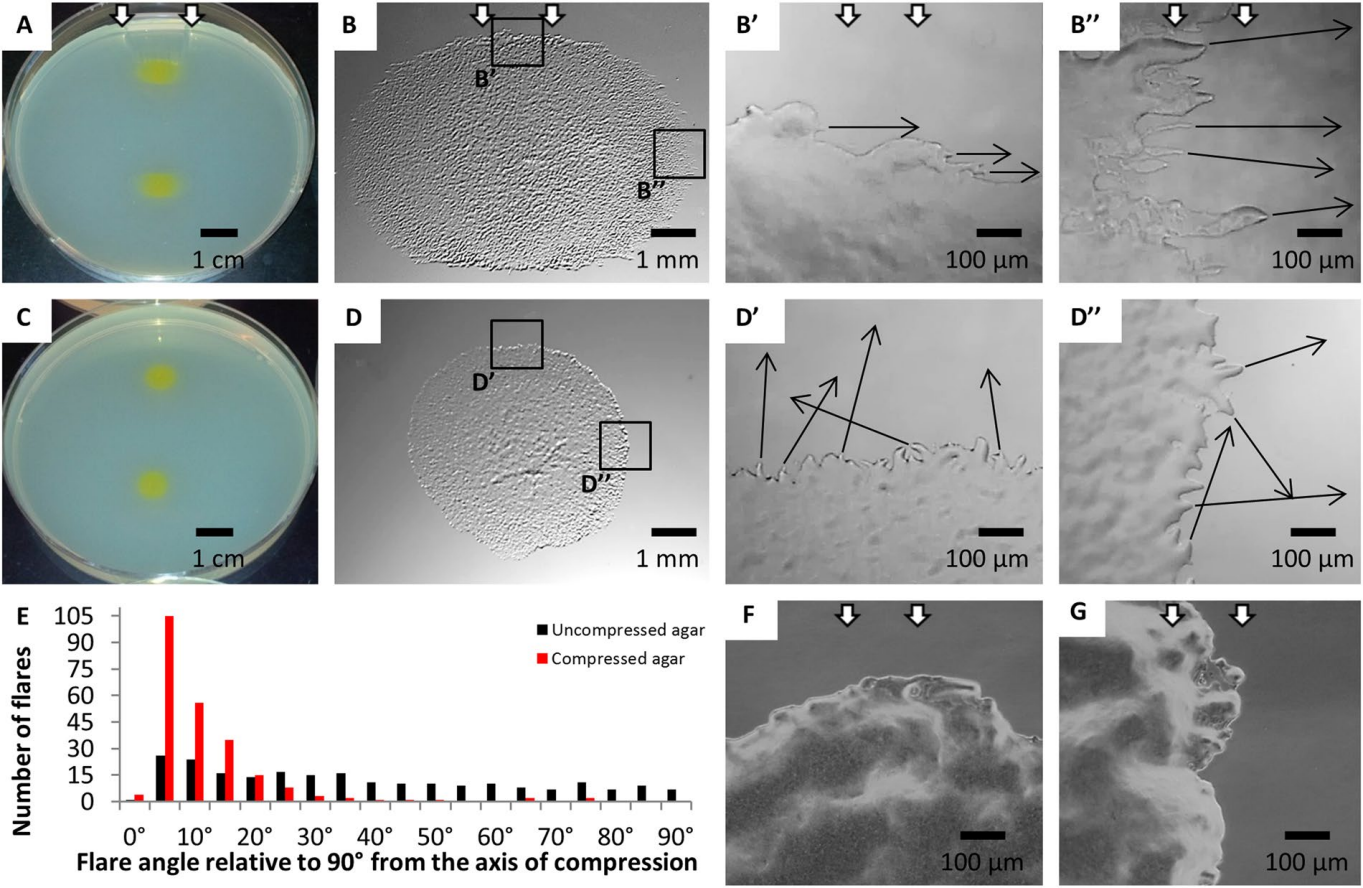
*M*. *xanthus* flares and colonies on compressed and uncompressed agar. Whole colony morphologies on compressed (**A**,**B**) and uncompressed agar (**C**,**D**) are shown. The white arrows in panels A, B, B’, B”, F, and G indicate the direction of compression. The direction of movement of selected flares or groups of cells (black arrows) at one edge of the long axis and short axis of a colony on compressed agar is shown in B’ and B”, respectively. The direction of movement of selected flares (black arrows) at the corresponding positions on uncompressed agar is shown in D’ and D”, respectively. The orientations of flares of four colonies incubated on uncompressed (228 flares, 22.4% within 10° of parallel with X axis) and compressed (235 flares, 70.2% within 10° of parallel with X axis) agar for 24 hours are shown in (**E**). The edges of the A^−^S^+^ colony on compressed agar shown in (**F**,**G**) are located at positions similar to those in B’’ and B’respectively. 96 hour old colonies shown in (**A**,**C**), 24 hour old colonies shown in (**B**,**D**,**F**,**G**).

We have been examining how bacteria respond to deformations of polysaccharide substrates to better understand how the physical characteristics of secreted polysaccharide affect bacterial behavior. For this study, we focused on *M*. *xanthus*, which was previously shown to respond to changes in the physical state of agar^7^. In particular, when agar is compressed by inserting a small plastic tube between the wall of a petri dish and the side of the agar slab^7^, the movements of groups of *M*. *xanthus* cells at the edge of the colony, known as flares, and the morphology of an expanding colony change. The images in Fig. 1A–D” show the morphologies of wild-type *M*. *xanthus* colonies after 24 hours on compressed and uncompressed agar. Colonies close to the inserted plastic tube expand primarily perpendicular to the direction of compression and have an elliptical shape (Fig. 1A and B), whereas colonies in a similar position on uncompressed agar expand radially outward and are round (Fig. 1C and D).

Additionally, the orientations of flares at the edges of colonies are different on uncompressed and compressed agar. On uncompressed agar, flares extend outward from the edges of colonies with no preferential orientation (Fig. 1D’ and D”) and only 22.4% are oriented roughly perpendicular (90° ± 10°) to the axis of compression (Fig. 1E). Furthermore, a given flare seldom maintains its orientation over a 12-hour period (Supplementary Video S1 and S2). Instead, the flares often turn to merge with the advancing colony front or join an adjacent flare. In contrast, 74.0% of the flares advancing outward from the edges of colonies on compressed agar are oriented 90° ± 10° to the axis of compression (Fig. 1B’,B” and E) and the flares tend to maintain this same overall orientation over 12 hours of time-lapse imaging (Supplementary Video S3). Furthermore, when a rare flare emerges from a colony on compressed agar in an orientation that is not perpendicular to the axis of compression (Fig. 1B’, Supplementary Video S4), it will typically reorient and start moving perpendicular to the axis of compression relatively quickly (Supplementary Video S4). As previously observed^7^, the colonies of an *M*. *xanthus* strain that is defective for A-motility are round on compressed agar and lack flares that are oriented perpendicular to the axis of compression (Fig. 1F and 1G), indicating that flare orientation and directed colony expansion are active processes that require an intact A-motility system.

**Figure 2:**
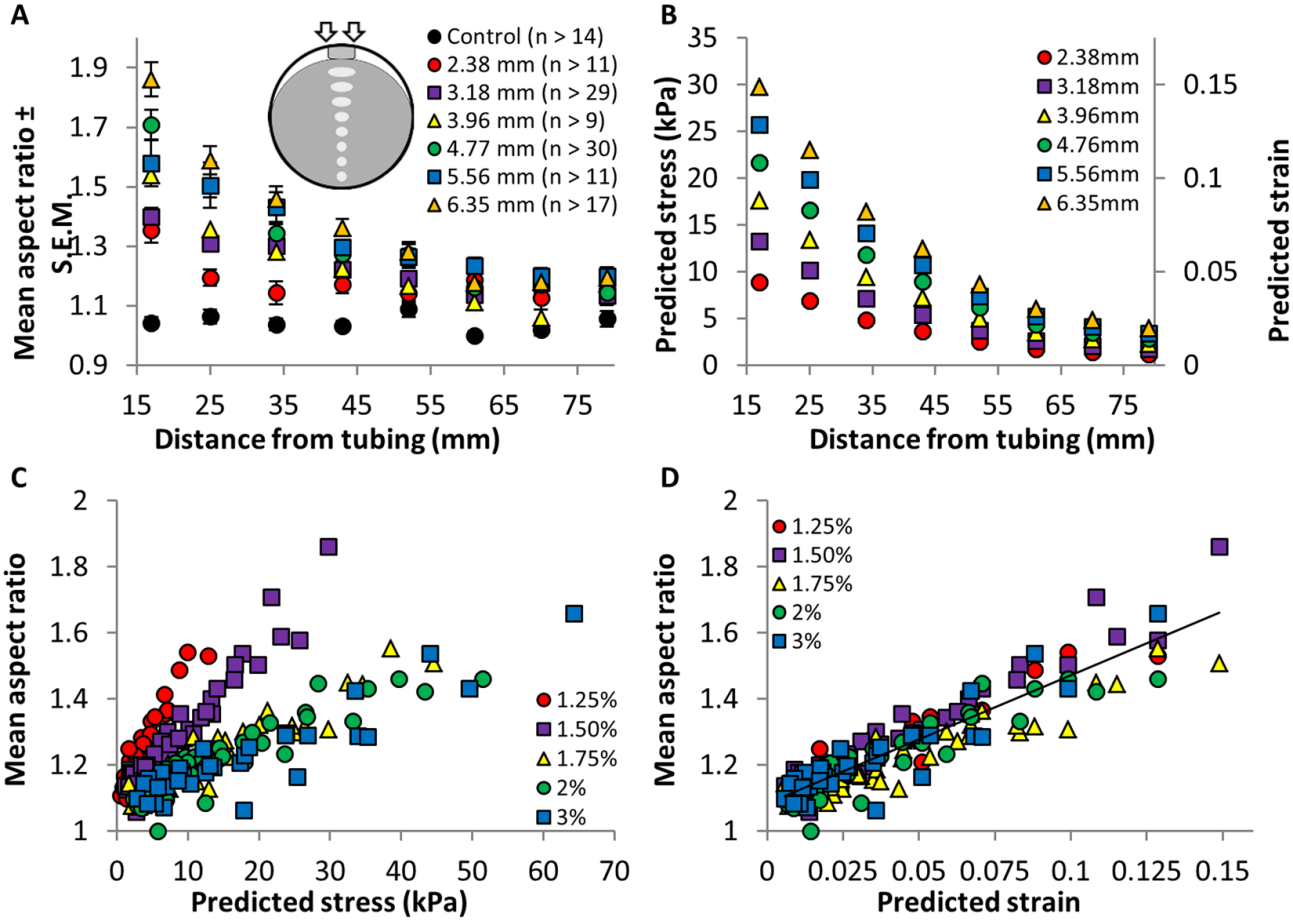
Effects of different levels of agar compression on aspect ratio, stress and strain. *M*. *xanthus* cells were spotted in a column down the centerline of compressed agar (inset in **A**) and the mean aspect ratios (±S.E.M.) of the colonies were determined (**A**). (**B**) Shows the predicted stress and strain at various distances from the inserted tubes. (**C**) and (**D**) show mean aspect ratios as a function of stress or strain for different agar concentrations.

To quantify the effect of agar compression on the colony morphology we determined the aspect ratios of colonies as a function of the distance from the inserted tube and the tube diameter that controls the degree of compression. Our experimental design is shown in Fig. 2A. In particular, eight drops of *M*. *xanthus* cells were placed in a column down the centerline of a compressed (via a 2.36 mm diameter tube) agar slab, which is in a circular petri dish, using a multichannel pipettor. The aspect ratios of colonies near the tube are relatively large and decrease as the distance between colonies and the tube increases. We find similar relationships between aspect ratio and distance from the tube when the tubes have different diameters, which keep the agar under different levels of compression (Fig. 2A). However, the larger the tube’s diameter, the more exaggerated this relationship becomes; the larger diameter of the tube, the larger the aspect ratio of the colony closest to the tube and the greater the distances from the tube before the aspect ratios approach that of colonies on uncompressed agar. Together, these findings indicate that *M*. *xanthus* is able to respond to different levels of agar compression. Interestingly, *M*. *xanthus* responds to compression-induced changes in the agar over relatively large distances; the aspect ratio of colonies that are about 60 mm away from the inserted tubes are still greater than those of colonies at comparable locations on uncompressed agar (Fig. 2A).

### Directed *M*. *xanthus* movements correlate with predicted strain

The assays described above were performed after the compressed agar plates were incubated overnight, which allowed the water that had accumulated on the surface to evaporate; this eliminated the possibility that the directed colony expansion followed the bands of water that accumulated on the surface after the agar was compressed. To identify a physical property of compressed agar that changes in step with colony aspect ratios we first examined the topography of compressed agar, as changes in the surface features of a substrate are known to affect the behavior of bacterial cells (for examples see refs 8–10). Phase contrast and 3D digital microscopy did not, however, reveal discernable changes in surface features such as wrinkles or troughs after compression of the agar substrate (Supplementary Figure S1; ref. 7). Furthermore, when we created troughs in the agar surface using grooved polyvinyl, we could detect the troughs and, as previously reported^11–15^, groups of cells followed the troughs as they moved (Supplementary Figure S1). Taken together, these findings suggest that the previously observed surface movements were not due to grooves or other compression-induced changes in agar topography.

We next examined the relationship between the stress and strain in compressed agar and changes in the aspect ratios of colonies. Specifically, we assumed that the agar slab behaves like a linear elastic medium, of known elastic constants, and used Comsol’s solid mechanics module to calculate the stress (force per unit area applied to the material) and strain (relative change in shape or size) profiles of a circular agar slab compressed by a tube of a given diameter, in agreement with our previous experimental set up (Fig. 2A). Since we operated under the assumption of linear elasticity, the predicted stress and strain within the agar are directly proportional to each other, and each can be converted to the other using the known elastic constant for each agar concentration^16^. The predicted stress and strain at positions on the agar that correspond to where the cells were spotted in previous experiments are shown in Fig. 2B. Similar to the colony aspect ratios (Fig. 2A), the predicted stress within the agar and predicted strain are largest in the regions closest to the inserted tubes and decrease with an increase in distance from the tubes.

In the next set of experiments, we obtained additional colony aspect ratio vs. distance data sets from substrates with different agar concentrations and hence varying stiffness^16^ (Supplementary Figure S2). In these experiments, changes in the stiffness of agar were predicted to have differential effects on the magnitudes of stress and strain. For example, it was predicted that a large stress would correspond to a small strain on stiff agar and a small stress would correspond to a large strain on soft agar. Hence, these experiments allowed us to test whether changes in colony aspect ratios correlated better with either predicted stress or strain, which might in turn allow us to disentangle the effects of predicted stress and strain on colony aspect ratios.

Figure 2C shows mean aspect ratios plotted as a function of predicted stress; however, the data do not collapse onto a single line. When the same aspect ratio data is graphed using predicted strain rather than stress^16^, as shown in Fig. 2D, a clear linear correlation (r = 0.915, r^2^ = 0.838) between the strength of the response to agar compression and predicted strain emerges. Taken together, these results suggest that the deformation of the agar (strain), rather than the amount of stress within the agar, may dictate the previously observed changes in the orientation of individual bacterial cells, groups of bacterial cells, and colony spreading patterns^7^.

### Compression-induced strain aligns and packs polysaccharide fibers

**Figure 3:**
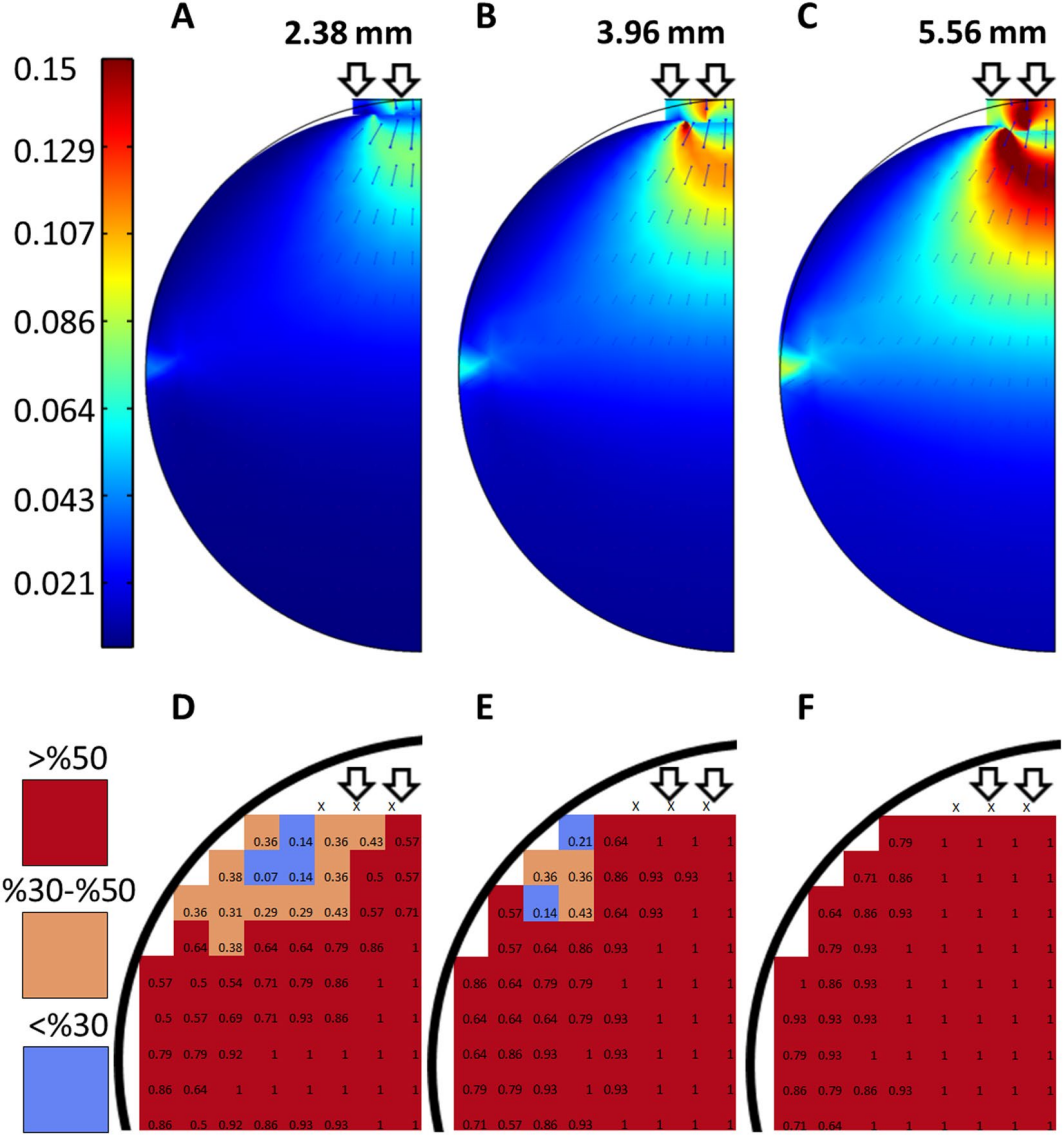
Predicted strain and birefringence of compressed agar. (**A**–**C**) Show heat maps of predicted strain due to compression of the agar substrate. (**D**–**F**) are heat maps of birefringence in regions of compressed 1.5% agar with high levels of predicted strain. The heat maps were generated by dividing the agar into grids and determining whether each square in the grid was birefringent after compression. The heat maps show the percentage out of seven replicates that each square in the grid was birefringent after compression. The diameters of the tubing inserted between the agar and the petri dish are shown above the heat maps.

It may be that the strain imposed by compression of agar increases the alignment of the matrix of polysaccharide chains or fibers and bacterial cells respond by orienting their movements along the aligned fibers, as previously suggested^7^. To examine this idea, we determined whether regions of the agar with relatively high predicted strain after compression (see Fig. 3) are birefringent, which would indicate an increase in molecular order in these regions of the agar.

The distribution of strain across the agar, as predicted by our numerical simulations (Fig. 3A–C), varies with the size of the inserted tube. Larger diameter tubes hold the agar under greater degrees of compression and result in a relatively large maximum strain, as well as a larger region of the agar where at least some strain is predicted. As the diameter of the inserted tubes increases, the regions of the agar with predicted strain expand down the centerline moving away from the tube and across the axis perpendicular to the tube. As shown in Fig. 3D–F, regions of the agar with moderate to high levels of predicted strain are birefringent in most or all of our experiments. In contrast, the corresponding regions in uncompressed agar lack birefringence in all experiments. These findings suggest that compression-induced strain tends to increase the molecular order in the agar substrate.

The increase in molecular order in compressed agar might be due to the alignment of polysaccharide fibers in the agar gel. To test this idea we used small-angle X-ray scattering (SAXS) and gels generated from the polysaccharide κ-carrageenan. We selected κ-carrageenan as a proxy for agar for several reasons. First, κ-carrageenan is chemically similar and thought to be structurally similar to the major polysaccharide (agarose) in agar^17, 18^. Second, like agar and agarose, κ-carrageenan is birefringent when compressed (Supplementary Figure S3). Third, κ-carrageenan is capable of inducing asymmetric colony spreading upon compression (Supplementary Figure S3). Fourth, κ-carrageenan forms thin gels that are less prone to buckling and breaking than agar gels, which enabled us to analyze them via SAXS analysis.

**Figure 4:**
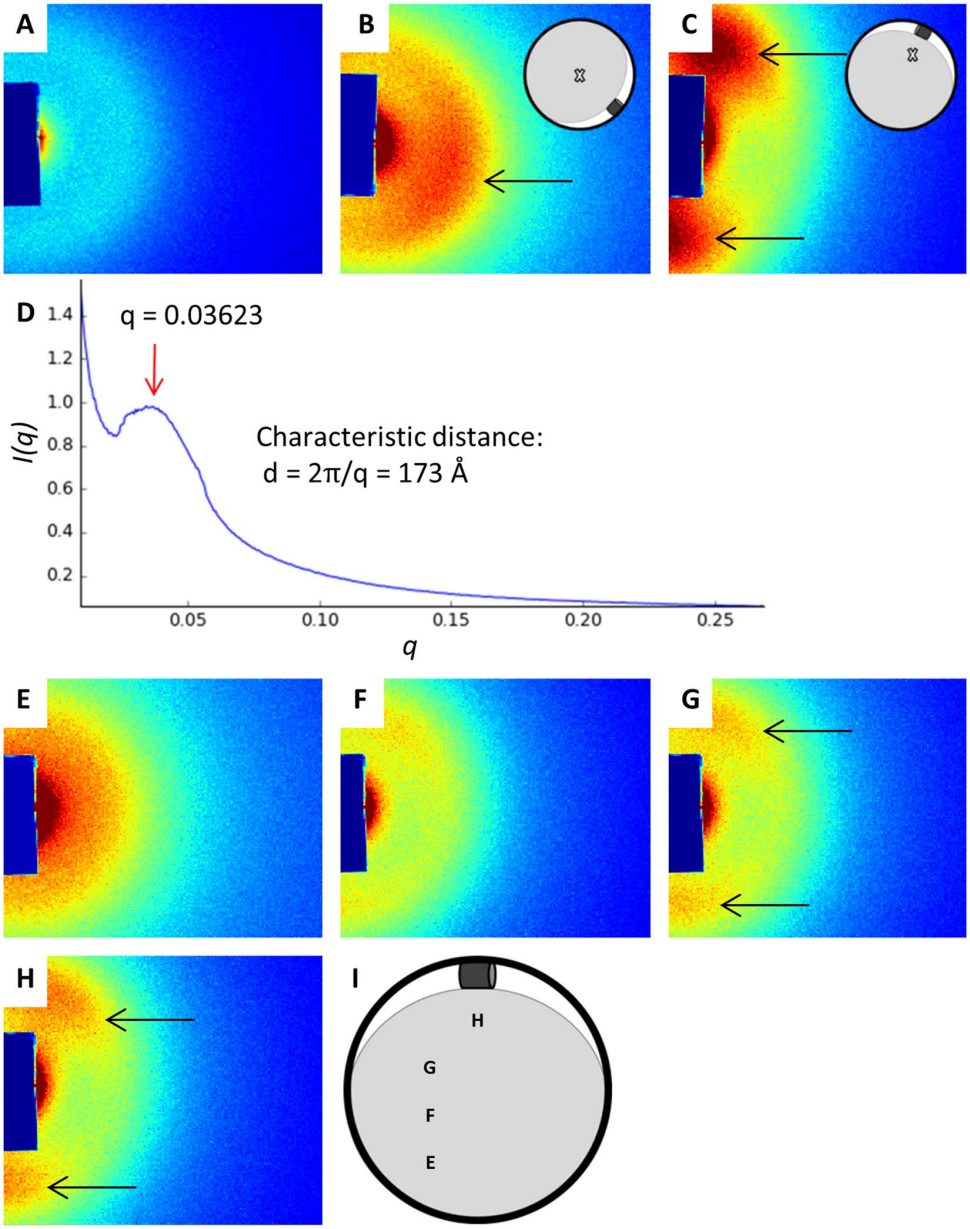
Small angle X-ray scattering (SAXS) analysis. The radially symmetric SAXS pattern before compression of the κ-carrageenan gel is shown in (**A**). The initial incidence of the X-ray beam and the corresponding SAXS pattern for the compressed κ-carrageenan gel are shown in (**B**). When the sample is rotated approximately 90°, as shown in (**C**), the SAXS pattern has two lobes of greater intensity (**C**). In (**D**), the plot of I(q) versus q shows X-ray intensity as a function of the scattering vector. The peak at q = 0.03623 (red arrow) indicates a characteristic distance of 173 Å between polymer bundles. (**E**,**F**) show the SAXS pattern when the incidence of the X-ray beam is shifted to different locations (shown in **I**) on the compressed κ-carrageenan gel. Black arrows point to the two lobes in the SAXS pattern. The colors shown in (**A**–**C**,**E** **–H**) indicate the relative detection intensity; redder colors represent greater intensity and bluer colors represent less intensity.

Before compression of the κ-carrageenan gels the SAXS pattern is radially symmetric (Fig. 4A), indicating the polysaccharide fibers in the gel are not inherently ordered or aligned. This finding is consistent with the birefringence data; κ-carrageenan gels lack birefringence and presumably molecular order before compression (Supplementary Figure S3). After compression, the SAXS pattern has two lobes of greater intensity that are apparent after the sample is rotated approximately 90° (Fig. 4B,C, for experimental set-up see Supplementary Figure S4). This SAXS pattern is characteristic of a nematic arrangement in which the long axes of molecules (in this case polysaccharide fibers) are aligned perpendicular to the axis of compression. The plot of I(q) versus q shows a peak at q = 0.03623 (Fig. 4D), which suggests a characteristic distance of 2π/q = 173 Å (17.3 nm) between aligned polysaccharide fibers. Hence, the polysaccharide fibers in the compressed κ-carrageenan gels seem to be aligned and tightly packed. Based on the SAXS patterns shown in Fig. 4, the alignment of the polysaccharide fibers is dependent upon the distance from the inserted tube that compresses the κ-carrageenan gel. In particular, the two-lobed SAXS pattern, which is indicative of the nematic alignment of the polysaccharide fibers, is detectable when the incidence of the X-ray beam is near the inserted tube but not when it is relatively far from the inserted tube. This relationship between the nematic alignment of polysaccharide fibers and the distance from the inserted tube that compresses the κ-carrageenan gel is similar to the relationship between colony elongation (Fig. 2A) and predicted strain (Fig. 3A–C) and the distance from the inserted tube that compresses agar gels.

### *M. xanthus* follows the long axes of aligned polysaccharide fibers

**Figure 5:**
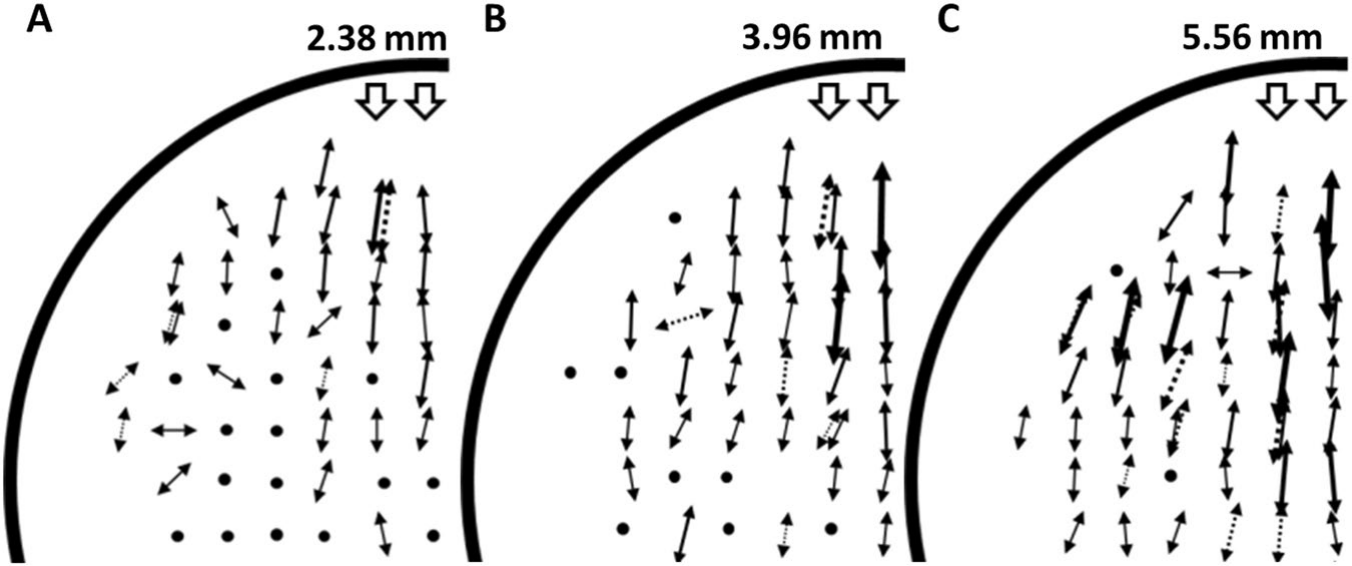
Surface spreading patterns at different locations on compressed agar. *M*. *xanthus* cells were spotted in regions of the agar that have moderate to high levels of predicted strain and aligned polysaccharide fibers. The arrows show the mean orientation of the short axes of 5–8 colonies (the long axes would be oriented 90° from the short axes) at each position and arrow length represents the relative mean aspect ratios. Dotted arrows represent data for colonies from comparable locations on the opposite side of the midline of the plate. Black circles represent mean aspect ratios below 1.1, similar to those observed on uncompressed agar. The diameters of the inserted tubing are noted above the diagrams.

To examine whether the previously observed changes in colony aspect ratios are due to the orientation of the polysaccharide fibers within the compressed agar substrate, cells were spotted in regions of the agar that have moderate to high levels of predicted strain and polysaccharide fiber alignment. It is notable that the aspect ratios tend to be large in the regions with moderate to high levels of predicted strain, and presumably some degree of polysaccharide fiber alignment, and similar to those on uncompressed agar in regions with little no predicted strain and polysaccharide fiber alignment (Figs 2A and 3A–C). In the regions of the agar with moderate to high levels of predicted strain, the long axes of the colonies are parallel to the predicted direction of polysaccharide fiber alignment and the short axes are perpendicular to the aligned polysaccharide fibers (Fig. 5A). As mentioned above, as the diameter of the inserted tube increases, the regions of the agar with predicted strain and polysaccharide fiber alignment expand down the centerline moving away from the tube and across the axis perpendicular to the tube. As shown in Fig. 5B,C, the regions of the agar slab where biased colony elongation is observed expands as the diameter of the inserted tube increases and the pattern of expansion is similar to that of predicted strain and polysaccharide fiber alignment.

Is tracking along the long axes of aligned polysaccharide fibers a common property in bacteria? In previous studies, four bacterial species responded to agar compression in a manner similar to that of *M*. *xanthus*; the colonies preferentially spread across the agar surface perpendicular to the direction of compression^7, 19–22^. This list of compression-responsive bacteria includes *Corallococcus exiguus*, which is a close relative of *M*. *xanthus*, and the distantly related firmicute *Bacillus mycoides*. To examine how widespread the response to agar compression and by extension the alignment and tight packing of polysaccharide fibers is, we spotted a variety of bacterial species onto plates containing compressed agar or κ-carrageenan and the appropriate growth media, and observed the colony morphologies (Table 1). In testing these bacteria, it became clear that a variety of species from several distant divisions of the phylogenetic tree are responsive to substrate compression. These bacteria have two common features: the individual cells are rod-shaped and they are capable of spreading across semi-solid surfaces. The vast majority of these bacteria have surface motility systems. However, the fact that *B*. *mycoides* (classified as non-motile) responds to substrate compression indicates that a surface motility system is not absolutely required. Among the surface motile bacteria, no specific motility system seems to be crucial, as species with different motility systems respond similarly. With few exceptions, the surface-motile bacteria that respond to substrate compression are capable of forming biofilms.

**Table 1:** Analysis of polymertropism in different bacterial species.

Species name	Phylum	Shape	Motile	Polymertropism	Reference	Source^a^	Gelling agent	Media	Temp.
*Alcaligenes faecalis*	Proteobacteria	Rod	Yes	Yes	This study	CBSC 154835 A	1% κ-carrageenan	CTTYE^57^	37 °C
*Bacillus cereus*	Firmicute	Rod	Yes	Yes	This study	CBSC 154870 A	1.5% agar	CTTYE	30 °C
*Bacillus megaterium*	Firmicute	Rod	Yes	Not observed	This study	NRRL B-14308	1.5% agar	CTTYE	30 °C
*Bacillus mycoides*	Firmicute	Rod	No	Yes, previously observed	This study, Stratford *et al*.^21^	NRRL B-14811	1.5% agar	CTTYE	30 °C
*Bacillus subtilis*	Firmicute	Rod	Yes	Yes	This study	NRRL B-354	1.5% agar	CTTYE	30 °C
*Corallococcus exiguus*	Proteobacteria	Rod	Yes	Previously observed	Stanier 1942^19^	—	2% agar	Dung^19^	Not provided
*Enterococcus faecalis*	Firmicute	Sphere	No	Not observed	This study	CBSC 155600 A	—	—	—
*Escherichia coli* K12	Proteobacteria	Rod	Yes	Not observed	This study	NRRL B-3707	—	—	—
*Escherichia coli* RP437/pRSH103	Proteobacteria	Rod	Variable	Inconclusive^b^	This study	D. Ren^57^	—	—	—
*Flavobacterium johnsoniae*	Bacteriodetes	Rod	Yes	Previously observed	Fontes & Kaiser 1999^7^	—	1.5% agar	PY2^58^	27 °C
*Klebsiella pneumoniae*	Proteobacteria	Rod	No	Not observed	This study	CBSC 155095 A	—	—	—
*Lactococcus lactis*	Firmicute	Sphere	No	Not observed	This study	CBSC 155610 A	—	—	—
*Micrococcus luteus*	Actinobacteria	Sphere	No	Not observed	This study	CBSC 155155 A	—	—	—
*Micrococcus roseus*	Actinobacteria	Sphere	No	Not observed	This study	CBSC 155160	—	—	—
*Mycobacterium phlei*	Actinobacteria	Rod	No	Not observed	This study	CBSC 155170 A	—	—	—
*Myxococcus xanthus*	Proteobacteria	Rod	Yes	Yes	This study, Fontes & Kaiser 1999^7^	—	1.5% agar	CTTYE	30 °C
*Paenibacillus alvei*	Firmicute	Rod	Yes	Yes	This study	NRRL B-383	0.5% κ-carrageenan	CTTYE	37 °C
*Paenibacillus dendritiformis*	Firmicute	Rod	Yes	Yes	This study	NRRL B-23299	1.5% agar	CTTYE	30 °C
*Paenibacillus phoenicis*	Firmicute	Rod	Yes	Yes	This study	NRRL B-59348	1.25% κ-carrageenan	ECM^59^	37 °C
*Proteus mirabilis*	Proteobacteria	Rod	Yes	Yes	This study	CBSC 155239 A	0.5% κ-carrageenan	CTTYE	37 °C
*Proteus vulgaris*	Proteobacteria	Rod	Yes	Not observed	This study	CBSC 155240 A	—	—	—
*Pseudomonas aeruginosa*	Proteobacteria	Rod	Yes	Yes	This study	CBSC 155250 A	0.5% κ-carrageenan	CTTYE	37 °C
*Pseudomonas fluorescens*	Proteobacteria	Rod	Yes	Not observed	This study	CBSC 155255 A	—	—	—
*Salmonella enteritidis*	Proteobacteria	Rod	Yes	Yes	This study	CBSC 155350 A	1% κ-carrageenan	CTTYE	37 °C
*Salmonella typhimurium*	Proteobacteria	Rod	Yes	Yes	This study	CBSC 155351 A	0.5% κ-carrageenan	CTTYE	37 °C
*Serratia marcescens*	Proteobacteria	Rod	Yes	Yes	This study	NRRL B-23389	1.5% agar	CTTYE	28 °C
*Shigella flexneri*	Proteobacteria	Rod	No^c^	Yes	This study	CBSC 155470 A	0.5% κ-carrageenan	CTTYE	37 °C
*Staphylococcus epidermis*	Firmicute	Sphere	No	Not observed	This study	CBSC 155556 A	—	—	—

^a^Carolina Biological Supply Co. (CBSC) or USDA Northern Regional Research Lab Culture Collection (NRRL) catalog numbers. ^b^The colonies did not show anisotropic spreading on 1.5% agar. On 1% and 1.25% κ-carrageenan anisotropic spreading was observed for some experiments and not others. ^c^ *Shigella flexneri* colonies had a slow expansion rate and did not show anisotropic spreading on 1.5% agar. On 0.5% and 1.25% κ-carrageenan the colonies expanded more rapidly and the colony expansion was anisotropic.

## Discussion

**Figure 6:**
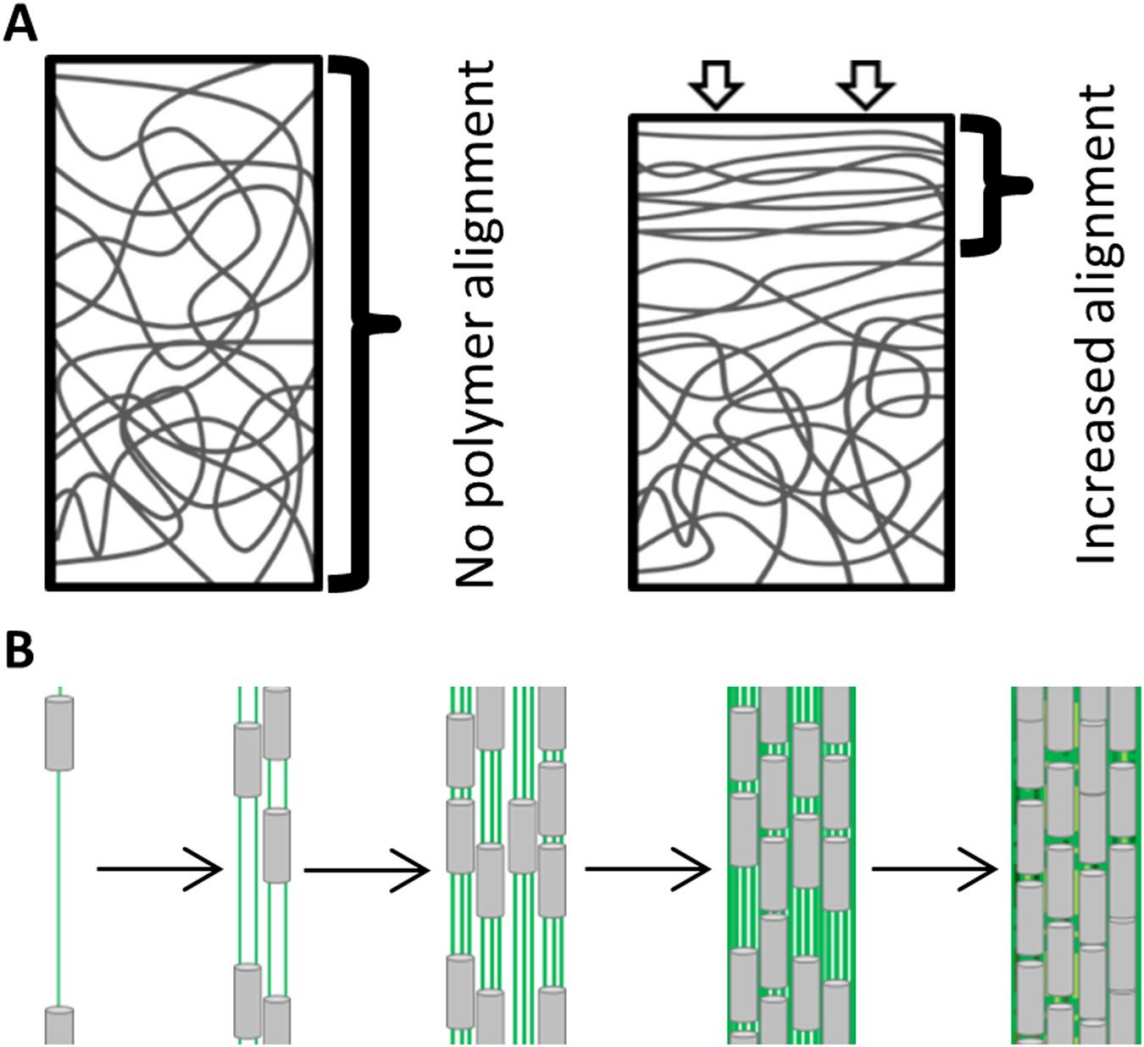
Model of compression-induced alignment of polysaccharide fibers and polymertropism. The diagram in panel A shows the unaligned mesh of polysaccharide fibers in an uncompressed gel and the increase in fiber alignment caused by compression. Based on the small-angle X-ray scattering (SAXS) experiments, polysaccharide fiber alignment and packing are greatest close to the source of compression where strain is predicted to be greatest. Cells (**B**, grey) extrude chains of polysaccharide, which are presumably aligned and form trails (**B**, green) that other cells follow. This behavior is reinforced over time (panel B left to right) as more cells move along the trails and deposit additional polysaccharide. Ultimately, this leads to alignment and coordinated movement of cells, and perhaps aggregate formation as previously suggested^45, 52^.

This work describes a physical change in the polysaccharide substrate of *M*. *xanthus* cells that induces an ordered collective behavior. Namely, when the polysaccharide fibers in agar are forced into alignment and to pack tightly via compression (see Fig. 6A), groups of *M*. *xanthus* cells change their movements to match the orientation of the long axes of the fibers. This ordered behavioral response, which we refer to as polymertropism, also leads to colony expansion primarily in the direction of the aligned polysaccharide fibers’ long axes. However, as noted below, we believe that polymertropism may play an important role in more complex emergent behaviors such as biofilm formation. Since the colonies of the majority (65%) of the rod-shaped, surface-motile, and biofilm-forming bacteria that we tested expand primarily along the long axes of aligned polysaccharide fibers (Table 1), we suggest that polymertropism might be a common behavior in bacteria that have these characteristics. In the case of *M*. *xanthus*, it is clear that surface motility is required for polymertropism, as the surface motility system known as A-motility is crucial for this behavior^7^. In the other cases, the link between surface motility and polymertropism has yet to be established; however, the fact that motility is known to be required for extensive surface spreading in most of these bacteria argues that motility is likely to be important for polymertropism in these organisms. It is notable that the variation in the mechanisms of surface motility among the polymertropism positive bacteria suggests that this behavior does not require a particular type of motility machinery. Indeed, this directed movement along the long axes of polysaccharide fibers may be analogous to movement of fibroblasts along the long axes of collagen fibers^23–25^, which is driven by actin filament assembly and disassembly^26–28^. Polymertropism may also be related to the observed behavior of rod-shaped bacterial cells in experiments performed with liquid crystals^29–32^. For example, when *Pseudomonas aeruginosa* cells are placed in a liquid crystalline matrix of concentrated DNA, they adopt the nematic alignment of the DNA chains^29^.

In *M*. *xanthus* polymertropism is driven by the A-motility machinery^7^, which includes focal adhesion complexes containing more than 14 proteins^33^. We suggest that the movement of *M*. *xanthus* cells along the tightly packed polysaccharide fibers in compressed agar helps focal adhesion complexes generate more traction force. In particular, we propose that the focal adhesion complex, which remains fixed to the substrate and drives the clockwise rotation and forward movement of the *M*. *xanthus* cell body^34^, is able to better grip (and slip less) when polysaccharide fibers are tightly packed. As a consequence, the A-motility machinery would be able to generate more force when *M*. *xanthus* cells move on the tightly packed polysaccharide fibers and the cells would make significant advances on the agar surface as they move along these fibers. Presumably, the alignment of the polysaccharide fibers would tend to keep the cells moving on the same general trajectory perpendicular to the axis of agar compression. It is also possible, as previously suggested, that movements along polymer fibers helps cells communicate over relatively long distances via mechanical signaling (polymer deformation), which would in turn facilitate coordinated group movements^35–39^.

Polymertropism may play an important role in the previously observed bacterial behavior of “slime trail” following. In particular, previous studies of surface motile bacteria showed that extracellular polysaccharide or “slime” is deposited when the cells move across their substrates^40–46^, and that other cells tend to follow these trails when they encounter them^45, 47, 48^. It has been suggested that the polysaccharide that forms the trails is extruded through pores or nozzles^41, 42, 49^ and, presumably, the process of extrusion through the relatively narrow central channel of such pores would align the polysaccharide and cause it to be tightly packed^41, 42, 50, 51^. We propose that individual and groups of cells follow the trails because of the aligned and tightly packed polysaccharide fibers. Presumably, the combination of trail deposition and trail following can produce the alignment and coordinated movement of large groups of cells in the absence of a chemical signal.

What role does polymertropism and slime trail following play in the behavior analyzed here? As noted above, slime is deposited in the direction of the bacterial surface movements and presumably the polysaccharide in slime is aligned and packed tightly when extruded. Therefore, the pioneer bacteria that move outward from a colony would follow the aligned and tightly packed polysaccharide in compressed agar and also deposit slime trails in the same orientation as the aligned fibers in the compressed agar. Other bacteria emerging from the colony would follow the trails if they encounter them. Hence, the compression-induced alignment and tight packing of the polysaccharide fibers in agar originally orient the bacterial surface movements and this directed movement is reinforced by the polysaccharide in slime trails, which is deposited on the surface of the agar and in the same orientation as the polysaccharide fibers in compressed agar.

We suggest that polymertropism (via slime trail following) may play a role in the formation of aggregates or groups of cells, which is a crucial step in a number of emergent behaviors including bacterial biofilm formation. As depicted in the model in Fig. 6B, cells in a biofilm deposit trails of aligned and tightly packed polysaccharide on their substrate as the move, which other cells in the biofilm use to orient themselves and to move upon. As the number of cells that move on the trails increases, more aligned and tightly packed polysaccharide would be deposited and the trails would become larger, which would allow more cells to join the trails by a positive feedback loop. It is also possible that the movement of large groups of cells on the previously deposited trails would further align and pack the polysaccharide (via traction force), reinforcing this positive feedback loop. Ultimately, these behaviors would lead to the alignment and coordinated movement of large groups of cells and perhaps the formation aggregates of cells, as predicted in the studies of *P*. *aeruginosa* ^45^ and the simulations of Balagam and Igoshin^52^. Of course, polymertropism would be one of many signals that are needed to construct a mature biofilm. Indeed, chemical signals such as quorum signals are known to be important for the formation of aggregates of cells during biofilm formation^53, 54^ (for review see refs 55 and 56). The challenge for the future will be to determine whether polymertropism does indeed have a specific role in emergent behaviors such as biofilm formation and, if it does, how it works in conjunction with chemical signals and perhaps other physical signals to complete these and other highly complex behaviors.

## Methods

### Assaying the response to substrate compression

The bacterial response to substrate compression was assayed using a method similar to that previously described by Fontes and Kaiser^7^. Briefly, CTTYE^57^ nutrient broth, unless otherwise noted (see Table 1 for the type of broth used for each bacterium), containing various concentrations of agar or κ-carrageenan was poured into 8.5 cm round or 10 cm square petri dishes, allowed to solidify, and compressed using 1.5-cm pieces of Tygon tubing with a diameter of 2.38, 3.18, 3.96, 4.76, 5.56, 6.53, or 7.1 mm. The petri dishes were subsequently placed at 28 °C to allow the water that was squeezed out of the gels by compression to evaporate. After the water on the surface of the gels had evaporated, eight 4 μl drops of bacteria at density of 5 × 10^9^ cells/ml were placed in a column down the centerline of compressed gels using a multichannel pipettor such that the furthest spot was 1 cm from the edge of the petri dish. This was repeated multiple times, with the mean number of colonies used to calculate each aspect ratio being approximately 31 for 1.25% agar, 23 for 1.5% agar, 16 for 1.75% agar, 20 for 2% agar, and 18 for 3% agar. For assays characterizing colony orientation and aspect ratio at various locations on the gels, the gels were divided into grids and 4 μl drops of *M*. *xanthus* at a density of 5 × 10^9^ cells/ml were placed in each square on the grid. The same grid locations were used for birefringence assays. Cultures were incubated for one day at temperatures indicated in Table 1, and the colony length (perpendicular to the direction of compression) and width (parallel to the direction of compression) were measured. 10–14 colony aspect ratios were determined for each position on the surface of the gels. Round dishes were used for all *M*. *xanthus* assays, square dishes were used for assaying other species^58–60^.

### Still and time-lapse imaging

Flares were examined with an Olympus CK2 inverted microscope and colonies were examined with a SMZ-168 Series Motic stereo zoom microscope. Images were captured with a 3.0 megapixel AmScope USB ocular camera and Toupview image capture software. Time-lapse videos were generated by capturing one image per minute for the first 12 hours of growth. Substrate surfaces were examined with a Hirox KH-8700 3D digital microscope and with an AmScope T390A-PCS compound microscope. Grooved substrates in Supplementary Fig. S1 were scored with the grooved edge of a fragment of a vinyl LP record.

### Substrate deformation simulations

Agar compression was simulated under different loads for various agar concentrations to determine the mechanical and deformational state of the substrate characterized by stress and strain on the agar surface. The simulation was performed using the solid mechanics module provided by Comsol, which assumes the condition of linear elasticity. The assumption of linear elasticity for the range of deformations considered here is supported by experimental studies^16^.

The geometrical representation of the simulation is illustrated in Fig. 3A–C with a top-down view in accordance with the experimental set up in Fig. 2A. The loading condition reduces to contact between two cylinders of cross-sectional diameters 10 cm and 1 cm, corresponding to the dimensions of the agar and the compressing tube, respectively. The simulation was performed with boundary conditions with the displacements (2.38 mm, 3.18 mm, 3.96 mm, 4.76 mm, 5.56 mm, 6.35 mm) induced by the tube at the contacting surface and zero displacement at the other end of the agar where it was bounded by the dish. The material properties of the agar were specified by a Poisson ratio *ν* = 0.4 and a Young’s modulus *E* = 100 kPa, 200 kPa, 300 kPa, 400 kPa, 500 kPa for agar concentrations of 1.25%, 1.50%, 1.75%, 2.00% and 3.00%, respectively^16^. The Young’s modulus of the Tygon tube was chosen to be 7 GPa for practical purposes, whose presence was to provide the correct boundary conditions at the contacting surface under loading forces that induced the experimentally imposed boundary displacements.

The stress *σ*_*ij*_ and strain ${{\epsilon }}_{ij}$ tensors on the surface of the agar were computed by solving the force balance equation (1)

1 $${\partial }_{j}{\sigma }_{ij}=0$$

together with the constitutive equation (2)

2 $${{\epsilon }}_{ij}=\frac{1}{E}[(1+\nu ){\sigma }_{ij}-\nu {\delta }_{ij}{\sigma }_{kk}]$$

under the imposed boundary conditions. The principle directions of the stress tensors coincided with the short axis of bacterial colonies (Fig. 5). While the stress and strain tensors completely specified the mechanical and deformational state of the substrate, the goal was to construct a scalar quantity from the tensors that is proportional to the response of the bacteria to the anisotropic substrate deformation measured by the aspect ratio of the colony. For this, we chose the Von Mises stress, which was defined by equation (3)

3 $${\sigma }_{VM}=\sqrt{\frac{3}{2}{\sigma }_{ij}^{^{\prime} }{\sigma }_{ij}^{^{\prime} }},$$

with equation (4)

4 $${\sigma }_{ij}^{^{\prime} }={\sigma }_{ij}-\frac{1}{3}{\delta }_{ij}{\sigma }_{kk},$$

as the deviatoric part of the stress tensor. This scalar quantity was proportional to the magnitude of the deviatoric stress on the surface of the agar. In parallel, we defined a scalar quantity that was proportional to the magnitude of the deviatoric strain as in equation (5)

5 $${{\epsilon }}_{VM}=\sqrt{\frac{3}{2}{{\epsilon }}_{ij}^{^{\prime} }{{\epsilon }}_{ij}^{^{\prime} }},$$

which we termed as Von Mises strain. Using the relationship in linear elasticity defined in equation (6)

6 $${{\epsilon }}_{ij}^{^{\prime} }=\frac{1}{2G}{\sigma }_{ij}^{^{\prime} },$$

where (7)

7 $$G=\frac{E}{2(1+\nu )},$$

we have (8)

8 $${{\epsilon }}_{VM}=\frac{{\sigma }_{VM}}{2G}.$$

We recorded Von Mises stress and Von Mises strain obtained from the simulation along the middle line on the surface of the compressed agar (Fig. 2B).

### Birefringence

CTTYE broth with 1.5% agar was compressed using 1.5-cm pieces of Tygon tubing with a diameter of 2.38, 3.96, or 5.56 mm. Water that was squeezed out was allowed to evaporate. Gels in glass dishes were divided into grids and each square in the grid was examined between cross-polarized filters with an Olympus BX51 microscope fitted with a U-AN360P filter. Changes in birefringence were monitored visually as the sample was rotated between cross-polarized filters. The proportion of 7 replicates that were birefringent at each square on the grid was subsequently determined. Only the presence or absence of birefringence at each location was noted; the magnitude of changes in birefringence was not determined.

### Small angle X-ray scattering (SAXS)

SAXS data was collected on the G1 line at Cornell High Energy Synchrotron Source (CHESS). Briefly, a sheet of isotropic 25-um thick polystyrene (Goodfellow Cambridge Limited, England; model number LS437323 L O; ST311025/1) was taped onto a polyacrylic block with a circular hole such that the isotropic polystyrene was covering the hole. A black O-ring (outside diameter: 16.25 mm; inner diameter: 13.64 mm; thickness: 1.77 mm) was placed on top of the polystyrene sheet (Supplementary Figure S4). The liquid 1.5% κ-carrageenan solution was kept from gelling with a heated water bath before being transferred to the O-ring with a Pasteur pipet. Any air bubbles were removed via a pipette. To compress the gel, a piece of cut O-ring was inserted between the κ-carrageenan gel and the O-ring surrounding the gel.

## Electronic supplementary material

Supplementary Video S1.

Supplementary Video S2.

Supplementary Video S3.

Supplementary Video S4.

Supplementary Information
