# Supplementary material for: Polymertropism of rod-shaped bacteria: movement along aligned polysaccharide fibers
Source: Sci Rep. 2017 Aug 11;7:7643. doi: 10.1038/s41598-017-07486-0 (PMC5554183; doi:10.1038/s41598-017-07486-0)
Supplement: Supplementary file 5 — Supplementary Information [file 41598_2017_7486_MOESM5_ESM.pdf]

Polymertropism of rod-shaped bacteria: movement along aligned polysaccharide fibers

David J. Lemon[1], Xingbo Yang[2†], Pragya Srivastava[2‡], Yan-Yeung Luk[3*], Anthony G. Garza[1*]

[1]Department of Biology, Syracuse University, Syracuse, NY 13244

[2]Department of Physics, Syracuse University, Syracuse, NY 13244

[3]Department of Chemistry, Syracuse University, Syracuse, NY 13244

SUPPLEMENTARY INFORMATION

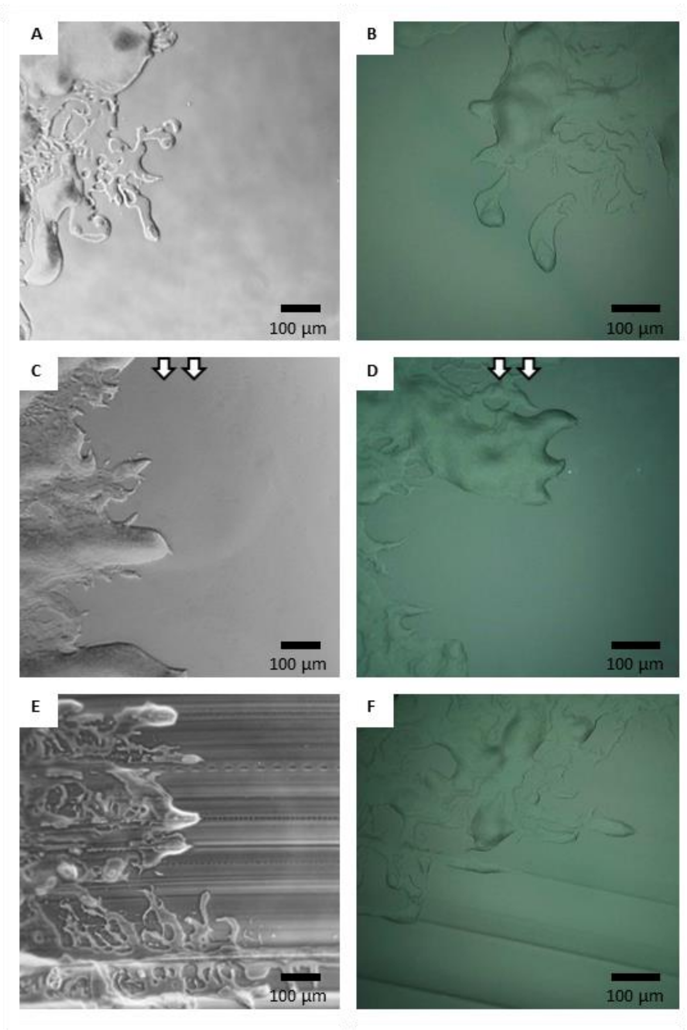

Supplementary Figure S1. Examination of substrate surface topography. Examination of the substrate surface at the *M. xanthus* colony edge on uncompressed (A-B), compressed from top to bottom (C-D), or uncompressed scored (E-F) substrates. Surfaces were examined with both phase-contrast (A, C, E) and 3D digital microscopy (B, D, F).

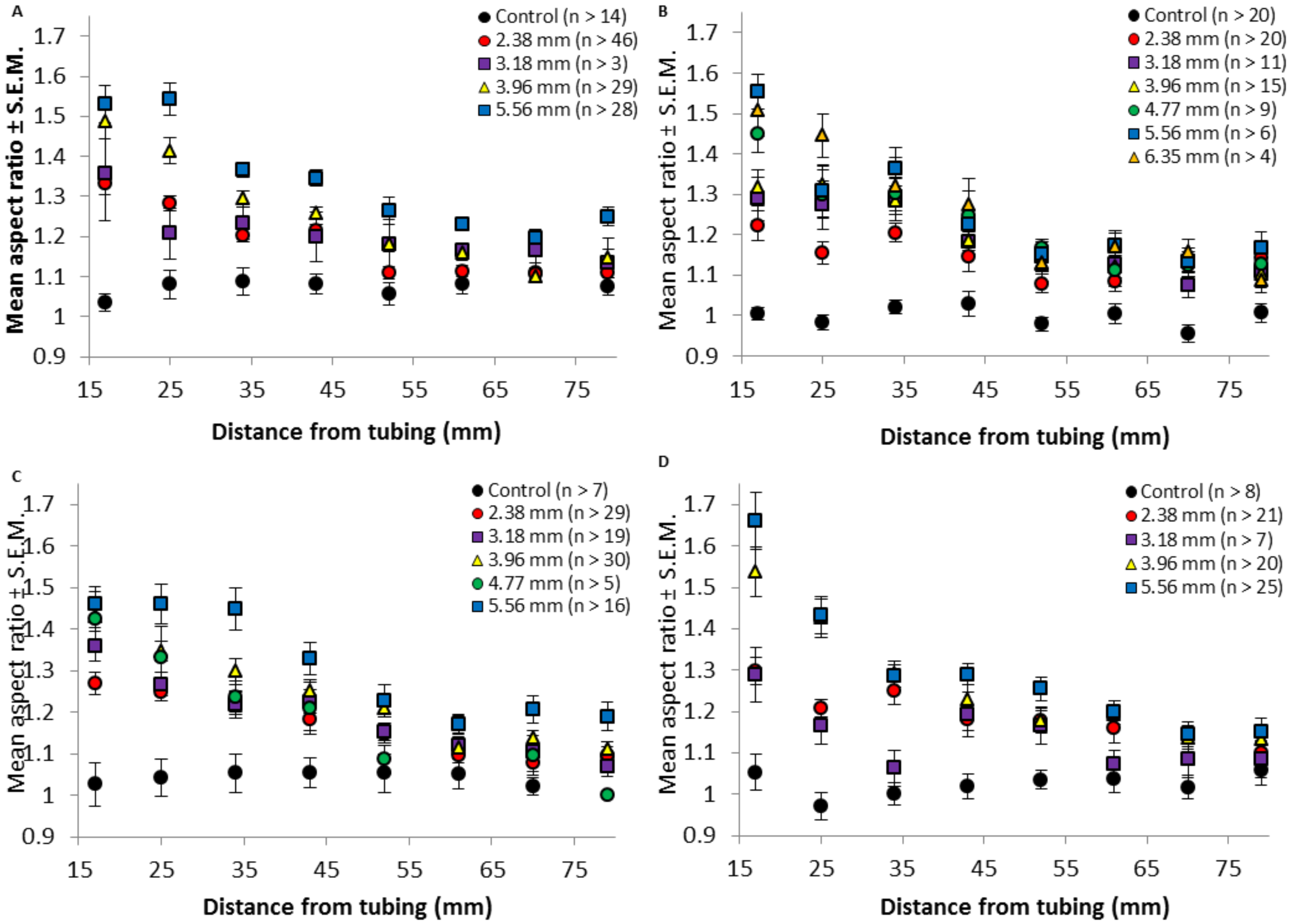

Supplementary Figure S2. Effects of agar concentration on aspect ratios. A-D show the mean aspect ratios (± S.E.M.) of *M. xanthus* colonies at various distances from inserted tubing with different diameters. The mean number of colonies used to calculate each aspect ratio is approximately 31 for 1.25% agar (A), 16 for 1.75% agar (B), 20 for 2% agar (C), and 18 for 3% agar (D).

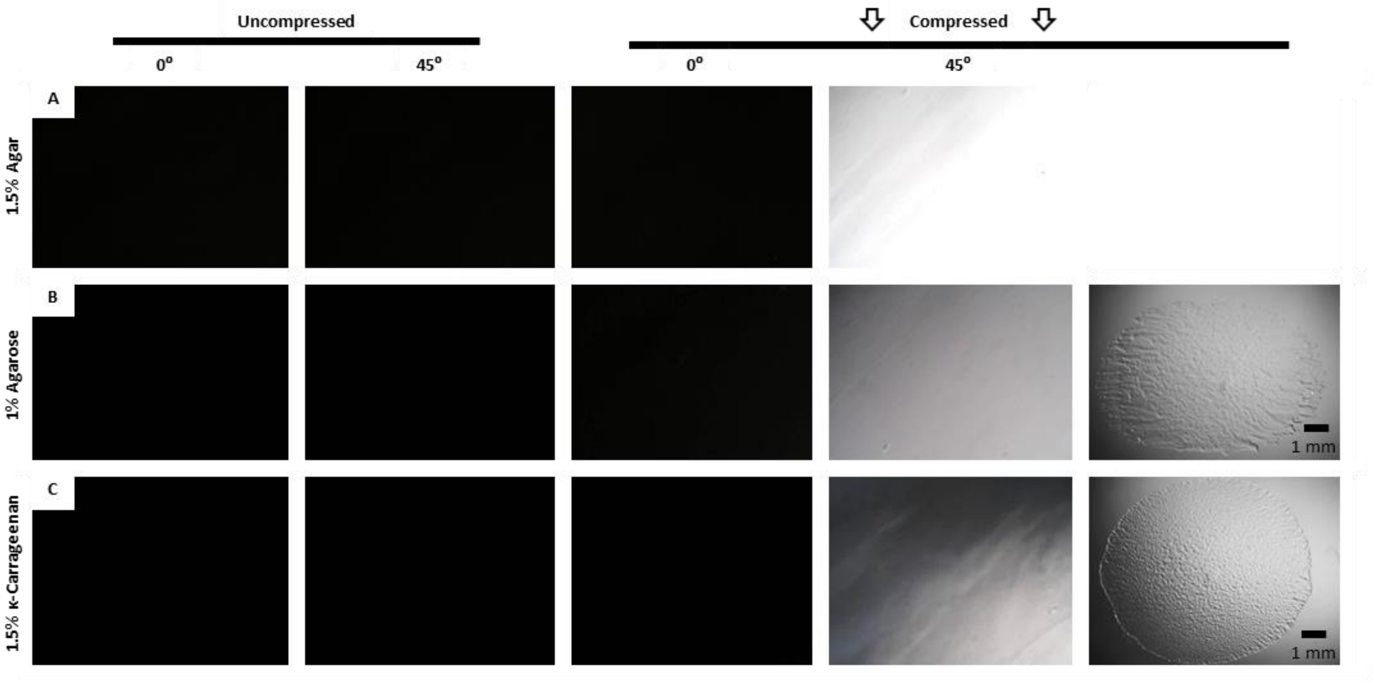

Supplementary Figure S3. Birefringence of agar, agarose, and κ-carrageenan.

Birefringence of 1.5% agar (A), 1% agarose (B), and 1.5% κ-carrageenan (C) gels before and after compression. The *M. xanthus* colony morphologies on compressed agarose and κ-carrageenan gels are shown in the right-most column.

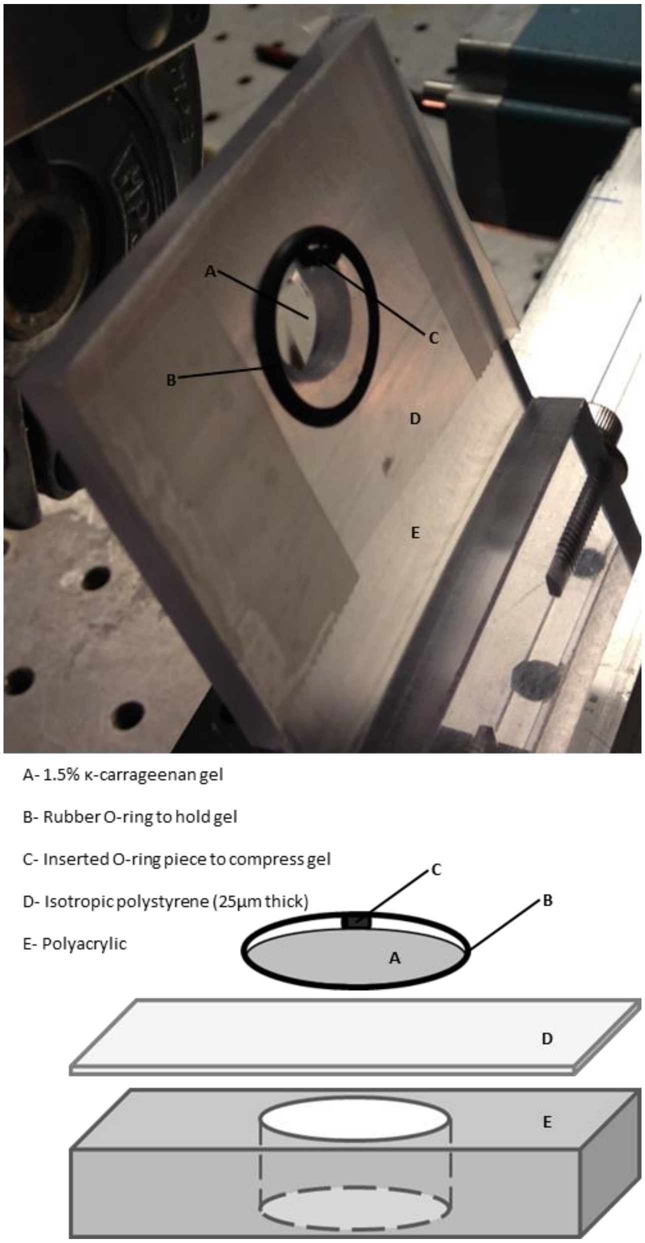

Supplementary Figure S4. The experimental design of the small-angle X-ray scattering (SAXS) analysis. The 1.5% κ-carrageenan gel (A) is held in position by an O-ring (B) and compressed by a piece of cut O-ring (C). The gel was placed on a 25 μm thick polystyrene plate (D), which was in turn placed over the hole in the polyacrylic support (E) such that the X-ray beam passed through the gel and the isotropic polystyrene, but not the polyacrylic support.

Supplementary Video S1. 12 hour time lapse of flare movements on uncompressed substrate at the edge of the *M. xanthus* colony's side. Flares move generally outward without clear orientation and are relatively short-lived. Comparable to Figure 1 D''. Video is 3.7 mm wide.

Supplementary Video S2. 12 hour time lapse of flare movements on uncompressed substrate at the edge of the *M. xanthus* colony's top. Flares move generally outward without clear orientation and are relatively short-lived, similar to Supplementary Video 1. Comparable to Figure 1 D'. Video is 3.7 mm wide.

Supplementary Video S3. 12 hour time lapse of flare movements on compressed substrate (compressed from top to bottom) at the end of the *M. xanthus* colony's long axis. Flares move generally rightward, are largely parallel with one another, and are relatively long-lived. Comparable to Figure 1 B''. Video is 3.7 mm wide.

Supplementary Video S4. 12 hour time lapse of flare movements on compressed substrate (compressed from top to bottom) at the end of the *M. xanthus* colony's short axis. Few flares emerge from the top, those that do move predominantly left or right. Comparable to Figure 1 B'. Video is 3.7 mm wide.
